# Health effects of caregiving and coping with severe mental disorders: A caregivers’ experience

**DOI:** 10.4102/sajpsychiatry.v30i0.2144

**Published:** 2024-03-29

**Authors:** Olindah Silaule, Fasloen Adams, Nokuthula G. Nkosi

**Affiliations:** 1 Department of Occupational Therapy, Faculty of Health Sciences, University of the Witwatersrand, Johannesburg, South Africa; 2 Division of Occupational Therapy, Faculty of Medicine and Health Sciences, Stellenbosch University, Cape Town, South Africa; 3 Department of Nursing Education, Faculty of Health Sciences, University of the Witwatersrand, Johannesburg, South Africa

**Keywords:** informal caregiver, informal carer, caregiving, positive coping mechanisms, negative coping mechanisms, caregiver health, caregiver well-being, mental disorder, severe mental disorder

## Abstract

**Background:**

Informal caregivers are an essential health resource in the care of persons with severe mental disorders, particularly in South Africa where access to mental healthcare services is limited.

**Aim:**

The study aimed to explore and describe the coping strategies used by informal caregivers and the specific health impacts they face in the context of severe mental disorders in South Africa.

**Setting:**

The study was conducted in Bushbuckridge municipality situated in the northeastern parts of Mpumalanga province, South Africa.

**Methods:**

A descriptive qualitative methodology was used to conduct semi-structured interviews with 12 purposefully selected participants. Audio-recorded interviews were translated, transcribed and analysed inductively on NVivo12 using reflexive thematic analysis.

**Results:**

The themes identified were caregivers’ experience of consequences of caregiving and caregivers’ experience of coping with their caregiving role. Participants experienced negative consequences on their emotional, mental and physical health. The participants use internal and external resources to cope with the challenges they face, and many highlighted using emotion-focused coping strategies.

**Conclusion:**

The findings revealed an urgent need to develop support strategies to strengthen informal caregivers’ coping and promote good health particularly in rural South Africa where informal caregivers play a crucial role in the management of severe mental disorders.

**Contribution:**

The finding demands that policymakers and healthcare providers prioritise the health and well-being of the informal caregivers. There should be policies targeted specifically at developing and implementing caregiver-orientated healthcare services.

## Introduction

Mental disorders are a leading cause of disability and significant contributor to the global disease burden.^1^ In lower- and middle-income countries (LMICs), mental disorders account for 5% of the total disease burden and 19% to the years lived with disability (YLDs).^2^ Severe mental disorders (SMDs) including schizophrenia, bipolar disorders, and depressive disorders are among the top 10 leading causes of disability within LMICs.^3^ In South Africa (SA), schizophrenia contributes approximately 2.1% to the overall disease burden,^4^ and bipolar mood disorders have been identified as affecting 4 million people, indicating that 1% of the population suffers from these disorders.^5^ A lifetime prevalence of depressive disorders is estimated at 9.8% and a 12-month prevalence at 4.9% making it a major contributor to the disease burden in the country.^6^

The growing burden of mental disorders in LMICs is exacerbated further by the limited access to mental health services.^7^ Limited funding, poor policy implementation, a lack of infrastructure, limited institutional support and staff shortages have been identified as leading factors contributing to the limited access to mental health services in LMICs.^7,8^ The treatment gap between those who need and those who receive treatment is estimated to be at 90% within LMICs.^9^ It is estimated that only 15% – 25% of people with mental disorders receive treatment in LMICs.^10^ In SA, only 27% of persons with SMDs receive adequate treatment.^11^ The limited access to mental health services calls for informal caregivers including the families, friends and relatives to become key role players in the care and management of SMDs.^12^

## The role of informal caregivers in severe mental disorders

The limited access to proper care, treatment and rehabilitation for people with SMDs requires informal caregivers (family, friends, or any person providing care without remuneration) to become a vital healthcare resource in LMICs including SA. The chronic nature of severe mental disorders often results in serious functional limitations that compromise their ability to independently fulfil roles as friend, family member or a worker.^13^ Subsequently, informal caregivers take charge of the physical, emotional, medical, and financial needs of care recipients with a SMD.^14^ The inherent tasks fulfilled by the informal caregivers in their role include taking care of the day-to-day needs of the care recipients including supervising participation in self-care activities, providing meals and safety for the mental healthcare user (MHCU), seeking help for the MHCU, early identification of relapse, supervising medications, and looking after their financial needs.^12,15^ In addition, informal caregivers are required to meet the emotional needs of the MHCUs and deal with the unpredictable, hostile, or risky behavioural disturbances exhibited by the MHCUs.^16^ Consequently, fulfilling the role of caregiving tends to be demanding and often introduces an intolerable strain.^17^

## Negative consequences for caregivers

The role of informal caregiving is a mammoth task and many are thrust into this role without preparation, knowledge or support.^14^ Studies conducted in LMICs revealed a general neglect of the informal caregivers’ health status.^14,18^ This is because most of the attention is directed towards the health and well-being of the MHCUs.^19^ Informal caregivers are often excluded from the decision-making processes regarding the mental disorder and treatment, and their needs are not taken into consideration; as a result, these caregivers do not have a chance to express their concerns and needs.^18^ Without preparation, knowledge or support, informal caregivers of persons with SMDs often experience changes in their life.^14^ Caregiving is associated with several negative experiences including physical, mental, and social consequences,^18,20,21,22^ which subsequently impact their quality of life.^23^ Previous studies conducted in LMICs reveal that caregivers experience negative emotions such as irritability, frustration, guilt, shame, and helplessness, which predispose them to mental health problems such as depression, anxiety, stress, and burnout.^14,22,24,25,26^ In addition, caregivers experience deterioration in their physical health, including developing physical conditions such as diabetes, high blood pressure, and body aches.^14,24,27^ Research evidence emphasises the importance of caregivers in the well-being and relapse prevention of persons with SMDs,^28,29^ and thus, any negative consequences on the caregivers’ health threaten the well-being of their care recipients.

## Coping mechanisms

Literature highlights that different informal caregivers utilise various coping mechanisms when caring for the MHCUs.^30,31^ Coping mechanisms relate to any cognitive and behavioural efforts made by any person to prevent, manage, or control emotional distress and protect them from the effects of a stressful life event.^32^ Lazarus and Folkman^32^ classify coping mechanisms into problem-focused and emotion-focused coping mechanisms. Problem-focused coping mechanisms use problem-solving skills to deal directly with a stressful situation, and emotion-focused coping mechanisms relate to efforts made to change one’s view about a stressful life event.^32^ These mechanisms can be positive or negative. Positive mechanisms include cognitive and behavioural responses such as positive thinking, information seeking, problem solving, and action-orientated pursuits, and social coping mechanisms include social support from family, friends, and religious organisations, access to community resources, and professional support.^33,34^ Some of the positive coping mechanisms used by informal caregivers are accessing and using available services, spending time on self-care related activities, sharing experiences with others, accessing information on mental disorders, building resilience, participating in religious activities, and relying on social support from family and friends.^24,33,34^ In their study, Ong et al.^30^ established that informal caregivers of persons with schizophrenia who utilised positive mechanisms such as social support, spirituality or religious coping, active coping, acceptance and positive reframing experienced lower levels of distress.

In contrast, negative coping mechanisms include avoidance behaviour,^33^ disregarding the care recipient, exhibiting resentment towards the care recipient, isolation, denial, and using medication to numb themselves.^25,34^ Negative coping mechanisms among the informal caregivers of MHCUs have been associated with high levels of distress.^30^ A study by Ata and Doğan^35^ revealed that informal caregivers of persons with schizophrenia were likely to use negative coping strategies including crying, denial of the MHCUs’ condition, self-blame, social withdrawal and diplaying aggressive behaviour towards the MHCU. The type of coping mechanism used has been linked with the informal caregivers’ level of education. In their study, Parks et al.^31^ established that informal caregivers who utilised positive mechanisms such as active coping and seeking information of the condition of the MHCU had higher levels of education, while negative mechanisms such as avoidant behaviour were common among those with lower levels of education.

## Research gap and study objective

Understanding the implications of caregiving on informal caregivers and the coping mechanisms they use are important to develop support to promote their health and well-being, which will in turn enable them to provide quality care to persons with SMDs.^34^ Previous studies conducted in LMICs, including South Africa, focused on the generic experiences of caregiving, and many other studies focused on the caregivers of persons with schizophrenia. Therefore, there is a paucity of evidence on the effects of caregiving on caregivers’ health and the coping mechanisms they use when caring for persons with SMDs. To address this gap, the study aimed to explore and describe the coping strategies used by informal caregivers and the specific health impacts they face in the context of severe mental disorders in South Africa.

## Research methods and design

### Study design

A qualitative descriptive research approach was used to conduct semi-structured interviews with informal caregivers of persons with SMDs between January 2022 and June 2022.

### Study setting

The participants were recruited from the first quantitative study that took place in the acute mental health unit in a district hospital in Bushbuckridge municipality, Mpumalanga province, South Africa. The mental health unit has a 50-bed capacity, which is the largest in the Mpumalanga province. This unit has a large catchment area as it offers both in- and outpatient mental health services to the residents of Bushbuckridge municipality and surrounding communities, and thus, covers a population of more than 546 215.^36^ Services provided include child, adolescent, and adult mental health services ranging from acute to chronic and forensic cases. Persons with SMDs are often treated and followed-up in this facility.

### Study population

Participants were purposely selected from the first quantitative study that aimed to establish the extent of caregiver burden among the informal caregivers of persons with SMDs.^37^ During the first study, potential participants who expressed interest were recorded. Once this study commenced, participants were contacted telephonically and invited to participate. A sample size of 12 participants was adequate to allow for full exploration of caregiving experiences and answering the research question. Data were considered saturated as no additional information were identified.

The primary inclusion criterion was that participants had to be an adult (+18 years) informal caregiver providing continuous unpaid care to a person with a SMD for longer than 6 months. Participants had to perform caregiving duties at least once a week. Only participants who presented with a subjective or objective burden (mild, moderate, or severe) in the first study were considered for participation.

### Data collection

Potential participants were contacted telephonically. The main researcher and the participants agreed on a date, time, and convenient place to conduct the interviews. All participants preferred for the interviews to be conducted in their homes, which provided adequate privacy and allowed for a rich exploration of experiences within the caregiving context. The main researcher is a local resident of Bushbuckridge municipality, and this made it easier to relate to the participants. Prior to the interviews, the researcher went through the information sheet detailing the purpose of the study. Informed consents, one for participation and another for audio recording, were gathered from the participants. Demographic characteristics of the informal caregivers were documented using a demographic questionnaire. An interview guide was used for the semi-structured interviews.^38^ To enable participants to share their experience from their own perspective, an open-ended question asked the participants to describe their experience of caregiving. Following this, the researcher asked questions aimed at exploring the burden of care among the informal caregivers. Participants were asked to describe the following: (1) the burdens encountered in their caregiving; (2) the impact of these burdens on their lives; (3) the sources of the burdens experienced; and (4) the resources and support they require in their caregiving role. The interviews lasted between 30 min and 60 min, depending on the experiences shared by the participants. All interviews were conducted in Xitsonga, the first language of both the participants and the researcher.

### Data analysis

All interviews were audio recorded. A language specialist working in a research facility in Bushbuckridge municipality transcribed and translated the interviews into English. Transcriptions were entered into NVivo 12 software and reflexive thematic inductive analysis was performed following the six-phase approach proposed by Braun and Clarke.^39^ Data familiarisation was carried out by listening to the recordings, and reading and re-reading the transcripts. Notes were taken that guided the generation of ideas for coding. Initial codes were assigned using inductive coding and each code was assigned a description to ensure consistency in the coding process. The codes were then grouped and initial themes were generated. Themes were developed and reviewed by generating maps on NVivo, which were printed and displayed to allow for comprehensive visual representation of the initial themes. At this stage, some codes became themes and additional codes were grouped for further analysis. The refining and renaming of themes were performed by reading coded extracts to ensure they merged coherently to form a pattern for a specific theme. This was followed by re-reading the dataset and familiarisation with the notes to ensure that no codes were missed and that the themes were valid. Trustworthiness was ensured by conducting an audit trail of the analysis process with an experienced qualitative researcher. The findings were then presented to research supervisors who reviewed the analysis process and by conducting peer briefings. This process allowed for reviewing, refining, and naming the themes as well as promoting the quality and truthfulness of the findings. The main researcher kept a reflexive journal to document all the thoughts and observations made during the study.

### Ethical considerations

The Human Research Ethics Committee of the University of the Witwatersrand (M200957) approved this study. Permission was also sought from the Mpumalanga Department of Health Research Committee (MP_202010_010). Informed consent was obtained from the participants before data collection to participate in the study and to record audio during the interviews. To ensure confidentiality, each participant was assigned a participant ID.

## Results

A summary of the informal caregivers’ demographic and caregiving characteristics is presented in Table 1. Most participants are the mothers (50.0%) of the mental healthcare users (MHCUs) and are above the age of 65 (41.7%). The majority (66.7%) live in the same residence as the care recipient and have been providing care for 11–20 years (58.3%). Most caregivers spend between 1 h and 8 h a day on caregiving (50.0%) and others spend 19 h – 24 h per day (41.7%).

**TABLE 1:** Demographic characteristics of informal caregivers of persons with severe mental disorders (*N* = 12).

Characteristic	Frequency	Percentage
**Age**		
20–39	3	25.0
40–65	4	33.3
+65	5	41.7
**Gender**		
Female	9	75.0
Male	3	25.0
**Marital status**		
Single	2	16.7
Married	7	58.3
Divorced	2	16.7
Widowed	1	8.3
**Education**		
Primary school level	2	16.7
Secondary school level	4	33.3
Tertiary level	2	16.7
Uneducated	4	33.3
**Employment status**		
Employed	1	8.3
Unemployed	4	33.3
Pensioner	6	50
Self-employed	1	8.3
**Relationships**		
Mother	6	50
Father	2	16.7
Sibling	4	33.3
**Number of care recipients**		
1	10	83.3
2	2	16.7
**Duration of caregiving**		
5–10 years	5	41.7
11–20 years	7	58.3
**Daily caregiving**		
1–8 h	6	50
9–18 h	1	8.3
19–24 h	5	41.7
**Monthly household income (ZAR)**		
≤ 1000	1	8.3
1100–5000	10	83.3
≥ 5000	1	8.3
**Monthly medical costs (ZAR)**		
≤ 100	1	8.3
101–500	11	91.7
**Residence**		
Same as care recipient	8	66.7
Different as care recipient	4	33.3

ZAR, South African Rand.

The participants were asked to describe their lived experience of providing care to a person with a SMD, and two themes were identified from the data during the inductive analysis, namely caregivers’ experience of consequences of caregiving, and caregivers’ experience of coping with their caregiving role. Each theme has sub-themes that describe the expressed experiences in more detail.

## Caregivers’ experience of the consequences of caregiving

When describing their experiences of the consequences of caregiving, the participants highlighted these sub-themes: emotional consequences of caregiving, walking on eggshells, mental consequences of caregiving, and physical consequences of caregiving.

### Emotional consequences of caregiving

The participants expressed living in a constant state of vigilance because of the MHCUs’ previous violent behaviour and unpredictability caused by their mental conditions. Despite the MHCUs being on treatment, the caregivers feared they will relapse and exhibit the same behaviours as displayed prior to treatment. One caregiver said:

‘Even when I am with him, I am very cautious because I am scared that at any time he can change and be that person he used to be before he started taking the treatment.’ (TBM79, Mother, age 73)

The participants grieved the loss of potential for their care recipients to lead independent lives, which leads them to subsequently lose hope and experience feelings of anguish. One participant explained it as follows:

‘When I was raising my children, I had hope that one day they will be able to look after themselves so that I can also have a break. So, instead things are just getting worse, and it makes me realise that I will never have a break until I die.’ (TBM04, Mother, age 65)

### Walking on eggshells

The participants explained that they feel they must tread gently around the MHCUs as they fear that any disagreement with the MHCUs will trigger their illness. As a result, the participants follow the MHCUs’ lead to avoid triggering their illness, for example:

‘If he does not want to talk and you insist on talking to him, it triggers him, and sometimes you may even have to take him to the hospital. So, as a caregiver I have to be always gentle with him in everything.’ (TBM48, Father, age 73)

Some participants fear the MHCUs they are taking care of and give in to their demands to maintain harmony within their homes. One participant explained that ‘it is easier when I agree with him because if I do not, he gets angry and bad things happen when he is angry.’ (TBM06, Father, age 64)

### Mental consequences of caregiving

The participants reported insomnia, a depressed mood, and memory problems. The stress of caregiving triggers sleep disturbances and forgetfulness as the participants are preoccupied with caregiving responsibilities, and as a result, their mental health is compromised. This was captured in the following comments:

‘There are days where I do not sleep at all at night, and I cry a lot.’ (TBM79, Mother, age 73)

‘Since I have been caring for him, I am very forgetful.’ (TBM 04, Mother, age 65)

Some of the participants also relive past traumas because of their caregiving. One participant had a recollection of painful past events:

‘I have been living a very difficult life for many years because even when I was married my husband abused me. I decided to separate from him and come back home with my children. I was hoping that life will be better, but it doesn’t get any better. The whole situation changed for the worst.’ (TBM04, Mother, age 65)

### Physical consequences of caregiving

Some participants reported that they have developed medical conditions and others reported that their pre-existing medical conditions have worsened because of the stress of providing care. Most participants reported being diagnosed with hypertension since providing care; for example: ‘I was always stressed and crying to a point where I was diagnosed with hypertension, and I have been put on treatment because of that’ (TBM125, Mother, age 45). A participant who suffered a stroke said:

‘I was told that my blood pressure was very high and I was at a high risk of a stroke. Even now, there is a part of my body, which is not yet fine. It is not functioning well, and I get tired very easily because of it.’ (TBM48, Mother, age 68)

Other participants reported weight loss that they attribute to their caregiving role. One caregiver said, ‘This is not my normal weight. I have lost weight due to all the problems that I have faced.’ (TBM79, Mother, age 73)

## Caregivers’ experience of coping with their caregiving role

The participants reported various coping strategies they use in their caregiving role that are described in the sub-themes: internal resources for coping and external resources for coping.

### Internal resources for coping

The participants rely on their resourcefulness and resilience to help them cope with caregiving. Some of the strategies used include acceptance of their situation, perseverance, being patient, exercising self-control, loving the MHCU, and socialising with others. Accepting their responsibility as a caregiver allows the participants to show unconditional love to the MHCUs, which enables them to develop tolerance for their disruptive behaviour. One participant explained:

‘Providing care for a mentally ill person is very difficult, but for as long as that love for him is still there, we survive, and that is why when we do not see him around, we make sure that we go around and look for him to bring him home.’ (TBM79, Brother, age 42)

Most participants use religion to cope and reported accepting their situation as something God destined for them. One participant explained that she resorts to prayer as she has found it helps improve the MHCUs’ condition: ‘My support comes from prayer because when he starts, I just pray and he gets better.’ (TBM48, Mother, age 68)

Having a sense of a collective experience in caregiving is another coping strategy the participants identified. They said that seeing other informal caregivers at the hospital during the MHCUs’ follow-up visits gives them a sense of comfort and universality as they realise they are not alone in their situation. One participant explained that ‘the only way to deal with this is to understand that other people are going through the same situation, and it is difficult for them.’ (TBM102, Mother, age 77)

### External resources for coping

Having access to medical and social support helps some participants cope with their caregiving. Participants who access medication and hospitalisation for the MHCUs indicated that this helped stabilise the MHCUs, thus making it more manageable to provide care: ‘The hospital has helped as well. He stayed in hospital, and he was given injections and other treatment until he was better.’ (TBM79, Brother, age 42). Other participants indicated that access to social worker services within the community helps them to cope. The social workers conduct home visits and arrange rehabilitation for the MHCUs with substance abuse problems, which make the participants feel supported, as one participant explained:

‘The social workers were visiting us from time to time. They suggested that he should be taken to a rehab centre hoping that maybe he will forget about the things that he smokes. That shows that I had the support I needed in caring for him.’ (TBM04, Mother, age 65)

Some participants receive social support from family, the community, and religious organisations. They indicated that they received financial and emotional support from their families, which gave them encouragement. One caregiver said:

‘I am getting support from my mother and my siblings. They always call to check how we are doing and to encourage me. They also sometimes give me money to help me care for him, and pay for the cars that we hire to take him to the hospital.’ (TBM98, Sister, age 38)

Other participants said their families help with caregiving duties, which gives them time to attend to other tasks: ‘That girl who just came in helps if I have to go somewhere. She is the one who looks after her and remind her to take her medications and bath.’ (TBM102, Mother, age 77)

Some participants also receive help from community members who prevent the MHCUs from engaging in disruptive activities such as substance abuse, reinforce medication compliance, and take the MHCUs to the hospital. One participant said, ‘People used to help me to hold him down and tie him so that we can take him to the hospital for the medications’ (TBM04, Mother, age 65). Participants also reported receiving support from the church through prayer and words of encouragement, which enables them to accept their caregiving situation. One participant said:

‘They pray with me and for us. After praying, there is a change, and I come home feeling free and calm and be able to accept whatever situation that may come.’ (TBM102, Mother, age 77)

## Discussion

The study highlighted the health effects that caring for persons with a SMD has on informal caregivers in rural South African and the coping strategies they use. This study’s findings revealed that caring for a person with a SMD has negative consequences on informal caregivers’ emotional, mental, and physical health. This finding is similar to that of Phillips et al.^40^ that caring for a person with a SMD negatively impacts the physical and mental health of caregivers. According to Ntsayagae et al.,^25^ caregivers of persons with SMDs experience negative emotions, such as feelings of hopelessness, emotional pain because of the level of care required by MHCUs, and helplessness as the SMD progresses. The participants’ experiences in this study are consistent with this and highlighted the impact of caregiving on caregivers’ emotional health. The participants also had varying experiences that compromise their emotional health; for example, they reported living in a constant state of vigilance because of the MHCUs’ previous violent behaviour and the unpredictability of their mental conditions. Their sense of helplessness and hopelessness was attributed to grieving the MHCUs’ loss of potential to achieve independence, subsequently resulting in emotional pain. Ntsayagae et al.^25^ highlight that helplessness and fear often go hand in hand. The participants in this study live in a constant state of fear as they are cautious of their actions to avoid triggering a relapse in the MHCUs. Subsequently, they adopt avoidant coping strategies such as agreeing with the MHCUs to maintain peace in their homes, which is similar to what is reported in previous studies.^41,42^

Brain et al.^43^ notice that 85% of caregivers of persons with treatment-resistant schizophrenia reported a negative impact on their mental health. Gupta et al.^29^ reveal that those caring for persons with schizophrenia experience sleep difficulties and insomnia. The findings in this study are consistent with these results, and also revealed that the caregivers experience memory problems, which could be attributed to preoccupation with caregiving responsibilities and the stress of caregiving.^44^ These findings highlighted that informal caregivers are at risk of developing anxiety and depression, emphasising the need to integrate health screening for caregivers into routine clinical care.^45^ The experience of reliving past traumas expressed by the participants can be seen as a predisposing factor to psychiatric conditions such as post-traumatic stress disorders. These findings are similar to those reported by Nuwara et al.,^46^ who establish that the stress of caring for persons with schizophrenia and bipolar affective disorders places caregivers at risk of post-traumatic stress disorders. Similar to the findings of previous studies,^24,47^ the stress of caregiving encountered by the participants in this study manifests as physical illnesses, with some reporting weight loss, high blood pressure, and cardiovascular conditions such as stroke. The effects of caregiving on emotional, mental, and physical health highlight the urgent need for routine health screening among the caregivers because it will facilitate early interventions to promote caregivers’ health and well-being, thus enabling them to provide quality care to MHCUs.

Most participants use emotion-focused coping strategies such as positive skills and emotions such as love, patience, self-control, acceptance, and perseverance to help them develop a sense of resourcefulness and resilience to adapt to the stress of their caregiving role. This finding is consistent with the benefit-finding theories for stress adaptation and coping, which indicate exhibiting positive emotions is crucial for fostering a variety of adaptive and durable personal resources important for developing resilience.^48^ The use of religious coping expressed by participants in this study is similar to the finding by Modise et al.^49^ that caregivers of MHCUs use prayer as a coping mechanism as they believe this helps with their challenges. In contrast to other studies, the participants in this study found hope in being part of a collective of caregivers who share similar experiences in their caregiving. This emphasises the need for caregiver support groups to create opportunities for sharing and facilitating the universality for caregivers to realise they are not alone in their stressful circumstances.^50^ The findings also revealed that some participants use problem-focused coping strategies to seek and use medical and social support. Access to medication, social worker services, and social support from family, the community, and religious organisations are external resources that help the participants cope. Previous studies reveal that problem-focused coping strategies are more effective at reducing distress and improved patient outcomes compared to emotion-focused coping strategies^18,51,52^ because problem-focused coping strategies deal directly with the root cause of the stressful situation, and thus, encourage the use of both internal and external resources to cope.^51,53^ In contrast, emotion-focused coping strategies use internal resources to cope.^53^ While some participants in this study have access to medical and social support, it is important to notice that these services are not readily available for all participants. Therefore, most participants rely on their internal resources to cope, predisposing them to negative health consequences. Evidence suggests that the coping strategies used by the caregiver impact MHCUs’ health, relapses, and readmissions.^54^ It is therefore important to focus on building internal and external resources to facilitate coping and promote the health and well-being of informal caregivers and MHCUs.

As a qualitative study that used purposive sampling, the findings of this study cannot be generalised as they may not be representative of the experiences of all informal caregivers in Bushbuckridge municipality. In addition, participants for this study were selected from the first quantitative study, thus excluding those who did not participate in this study. The strength of the study is that it explored the full range of caregiving experiences, allowing for reporting on the positive aspects of caregiving, which is different from previous studies that mostly report on the negative coping strategies used by caregivers.

## Conclusion

The study provides insights into the impact of caregiving on the emotional, mental, and physical health of informal caregivers and the coping strategies they use. The findings revealed that caregivers use their internal resources to appraise their caregiving experience; however, this was insufficient because informal caregivers were still susceptible to negative health consequences. The findings demand that policymakers and healthcare providers prioritise the health and well-being of the informal caregivers. Routine health screenings should be implemented in clinical practice to ensure early detection of health conditions, which will also facilitate early interventions. Policies in mental health should clearly outline specific caregiver-orientated services to ensure the planning and implementation of such services. Future research should conduct in-depth explorations of the coping mechanisms used by informal caregivers of persons with SMDs and the opportunities to strengthen these coping mechanisms to promote good health among informal caregivers.
